# Experiences of activity monitoring and perceptions of digital support among working individuals with hip and knee osteoarthritis – a focus group study

**DOI:** 10.1186/s12889-022-14065-0

**Published:** 2022-08-30

**Authors:** Elin Östlind, Eva Ekvall Hansson, Frida Eek, Kjerstin Stigmar

**Affiliations:** 1 Department of Health Sciences, Lund University, Lund, Sweden; 2 Dalby Health Care Center, Region Skåne, Sweden; 3 Skåne University Hospital, Lund, Sweden

**Keywords:** Osteoarthritis, Qualitative, Wearables, Behavior change techniques, Mobile health, Digital support, Fitness trackers

## Abstract

**Background:**

Mobile health (mHealth), wearable activity trackers (WATs) and other digital solutions could support physical activity (PA) in individuals with hip and knee osteoarthritis (OA), but little is described regarding experiences and perceptions of digital support and the use of WAT to self-monitor PA. Thus, the aim of this study was to explore the experiences of using a WAT to monitor PA and the general perceptions of mHealth and digital support in OA care among individuals of working age with hip and knee OA.

**Methods:**

We conducted a focus group study where individuals with hip and knee OA (*n* = 18) were recruited from the intervention group in a cluster-randomized controlled trial (C-RCT). The intervention in the C-RCT comprised of 12-weeks use of a WAT with a mobile application to monitor PA in addition to participating in a supported OA self-management program. In this study, three focus group discussions were conducted. The discussions were transcribed and qualitative content analysis with an inductive approach was applied.

**Results:**

The analysis resulted in two main categories: A *WAT may aid in optimization of PA, but is not a panacea* with subcategories *WATs facilitate PA; Increased awareness of one’s limitations* and *WATs are not always encouraging,* and the second main category was *Digital support is an appreciated part of OA care* with subcategories *Individualized, early and continuous support*; *PT is essential but needs to be modernized* and *Easy, comprehensive, and reliable digital support*.

**Conclusion:**

WATs may facilitate PA but also aid individuals with OA to find the optimal level of activity to avoid increased pain. Digital support in OA care was appreciated, particularly as a part of traditional care with physical visits. The participants expressed that the digital support should be easy, comprehensive, early, and continuous.

**Supplementary Information:**

The online version contains supplementary material available at 10.1186/s12889-022-14065-0.

## Introduction

Osteoarthritis (OA) is a chronic and common musculoskeletal disorder occurring frequently in the hips and knees [1–3]. Individuals with hip and knee OA often experience pain and reduced function of the affected joint [3–5] which may lead to reduced quality of life and reduced work ability [6, 7]. Hip and knee OA are also associated with an increased prevalence of comorbidities and premature mortality [8, 9].

There is ample evidence that physical activity (PA) decreases pain, improves physical function and health-related quality of life in individuals with hip and knee OA [10]. PA is defined as “*any bodily movement produced by skeletal muscles that results in energy expenditure*” [11]. For all adults, the World Health Organization (WHO) recommend at least 150–300 min of moderate intensity PA, or at least 75–150 min of vigorous intensity PA, or a combination of both during the week for substantial health benefits [12]. Doing some PA but not reaching the recommended levels is still better than no PA at all [13]. However, despite the recommendations and evidence showing the effect of PA, previous research has reported that most individuals with hip and knee OA are not physically active enough [13, 14].

Interventions using behavior change techniques have previously been shown to improve adherence to PA in the short-term [15, 16]. Behavior change techniques are defined as the smallest “active ingredient” in an intervention and supports the individual in the behavior change process [17]. Some of the most effective behavior change techniques to enhance adherence to PA have been found to be goal setting, self-monitoring of behavior, social support, and non-specific reward [16]. These and several other techniques are often incorporated in mobile Health (mHealth) interventions [18, 19] which has frequently been used in the last decade to promote PA in different populations [20, 21].

MHealth is a subsegment of electronic Health and encompasses the use of mobile communication devices such as smartphones, tablets, personal digital assistants, and wearable activity trackers (WATs) for digital health [22–24]. WATs are increasingly popular among users but also in research with eight published studies in 2013 and 199 in 2017 [21]. They are often used for self-monitoring of PA and can provide the user with prompts and feedback to an application (app) on the smartphone or tablet [21]. Commercially available WATs measure different aspects of PA such as steps, distance walked, intensity level and heart rate [21]. WATs have been used in interventions to promote PA and systematic reviews have shown that they can be effective in increasing PA levels in healthy adults [25], older adults [19], individuals with rheumatic and musculoskeletal diseases [26], and other chronic diseases [27]. Several studies have also shown a high short-term adherence to WAT-use among participants in PA interventions [26, 28–31]. Other types of digital health are also used to support individuals with hip and knee OA. There are several examples of web-based platforms and mobile apps that offer digital support such as information, exercises, and feedback [32–34].

Before implementing new methods to promote PA and health, it is important to gain information about the users’, i.e. patients’, perceptions and opinions about the method [35]. Several published studies have reported experiences and perceptions of using digital solutions and mHealth to support self-management in adult arthritis and OA patients [36–42]. The experiences differ but, in general, the results showed that the digital solutions could aid in self-management, increase adherence to exercise and improve the patients’ communication with health care personnel. Apprehensions towards the digital solutions and wanted features of the digital support were also reported [36–42]. Only a few studies have reported on participants’ experiences of self-monitoring PA with a WAT [36, 42] and, to our knowledge, there are no studies on a Swedish, working age population. The results could add relevant information about OA patients’ experiences and perceptions of this area which might guide clinicians and researchers when designing and providing future OA care.

The aim of this study was to explore the experiences of using a wearable activity tracker to monitor physical activity and the general perceptions of digital support in OA care among individuals of working age with hip and knee osteoarthritis.

## Methods

### Design

We conducted a focus group study and applied qualitative content analysis to the data [43–46]. The consolidated criteria for reporting qualitative research (COREQ) were used as a guidance when reporting the study [47].

### Setting

This study was a part of a larger project investigating the effect of self-monitoring PA with a WAT in working individuals with OA [48]. The primary outcome in the C-RCT was work ability and the secondary outcomes were PA and work productivity. Briefly, a cluster-randomized controlled trial (C-RCT) was conducted with one control group (*n* = 74) and one intervention group (*n* = 86). Both groups received information about OA, self-management, and exercise in group lectures according to the Supported OA Self-management Program (SOASP) [49, 50]. In addition, the participants in the intervention group used a WAT, Fitbit Flex 2, and the Fitbit-app for 12 consecutive weeks. The Fitbit Flex 2 device is placed in a wrist-worn small rubber band and measured distance, steps, time in different activity levels etc., which can be observed in the Fitbit-app [51]. The Fitbits had a default step goal of 10,000 steps per day that was changed to 7,000 steps per day. This was changed to make the step goal more achievable for the participants but also because previous research has reported that taking 7,000 steps or more per day was associated with lower risk of mortality [52] and has been shown to correspond to 150 min of MVPA per week [53]. The participants were asked to monitor their activity daily, and they also received some automatic feedback from the app. Feedback could be positive push notifications when they reached their step goal, reminders to move or different badges of PA accomplishment. The feedback was visible in the app or sent to the participant’s e-mail.

### Participants

In this study, a combination of purposive and convenience sampling methods was used [54]. Participants from the intervention group of the C-RCT that participated in 2019 (*n* = 57) were approached by email and asked if they were willing to partake in focus group discussions about their experiences of using the WAT and their perceptions of digital support in OA care. We chose to ask only participants that had taken part of the intervention in 2019 so that they would more easily recollect the intervention. Out of all contacted potential participants (*n* = 57), twenty individuals agreed to participate but two dropped out due to different unforeseen events. Three focus group discussions with six participants in each were held. The groups were settled based on the participants preferences of date and, in general, the participants were not familiar with each other.

### Process

The first author EÖ moderated each session and the co-authors KS (discussion one and three) and EEH (discussion two) assisted. All three researchers are female, registered physiotherapists (PTs) and have experience in qualitative research. EÖ had previously met the participants on one or several occasions. However, these meetings took place as a part of the research project, e.g., delivery of Fitbit or group lectures in the SOASP. The participants had received short information on e-mail about the study in conjunction with their informed consent. They signed the informed consent and brought it with them at the time for the focus group discussion. Each group discussion was carried out in the same manner. The participants were offered coffee and a sandwich upon arrival to the conference room and were able to get casually acquainted with the other participants. The participants, the moderator and the assistant sat around a table. Before commencing the discussion, the moderator started with a brief introduction. It was emphasized that the participants could feel secure in talking freely, express their experiences and that there were no ‘right’ or ‘wrong’ things to say. Participants were also asked not to pass on the information that emerged during the discussions. A questioning route was thereafter used with an opening question, introductory questions, key questions and ending questions [45]. The questioning route was designed before the focus group discussions and was applied on all three sessions without any changes (Additional file 1). The questions were mostly open-ended and designed to answer the aim of the study. Discussions between the participants were encouraged. Follow-up questions or questions that targeted a specific participant were asked when needed. Field notes were taken by the assistant. At the end of each session, the assistant verbally summarized what had been discussed during the focus group and the participants were allowed to comment on this. After each focus group discussion, the moderator and the assistant had a brief debriefing where they reflected on the content of the focus group discussion. The focus group discussions lasted between 60 and 75 min and were conducted in November–December 2019. The three discussions and the debriefings were audio-recorded and transcribed verbatim by EÖ. Participant demographics were collected prior to this study in conjunction with the C-RCT and are presented in Table 1.

**Table 1:** Participant characteristics and physical activity levels (IPAQ-SF categories)

Characteristics of participants	(*n* = 18)
**Women**	13
**Age in years, mean (SD)**	58 (6.0)
**Married or living with partner**	15
**Education (postsecondary)**	9
**Sedentary work**	9
**Regularly used a WAT**	9
**Most affected joint**	
Hip	7
Knee	11
**IPAQ-SF, categorical**	
Low	5
Moderate	4
High	7
*Missing*	*2*

*SD* Standard deviation, *WAT* Wearable activity tracker, *IPAQ-SF* International physical activity questionnaire – short form

### Data analyses

The data from the focus group discussions were analysed using qualitative content analysis and the inductive approach as presented by Elo and Kyngäs [43]. No themes or categories were identified in advance. We followed the three phases of the analysis: preparation, organizing and reporting. All three transcribed focus group discussions were seen as *a unit of analysis.* The transcribed discussions were read through several times by EÖ and KS to become familiar with the data. Thereafter, the data was anonymized and organized using the software program NVivo (released 2020). Open coding was conducted in NVivo, headings were written using *annotations,* and codes were thereafter created. Similar codes were grouped in sub-categories and similar sub-categories were grouped in main categories. The process was not linear, and data was re-organized several times.

## Results

**Fig. 1:**
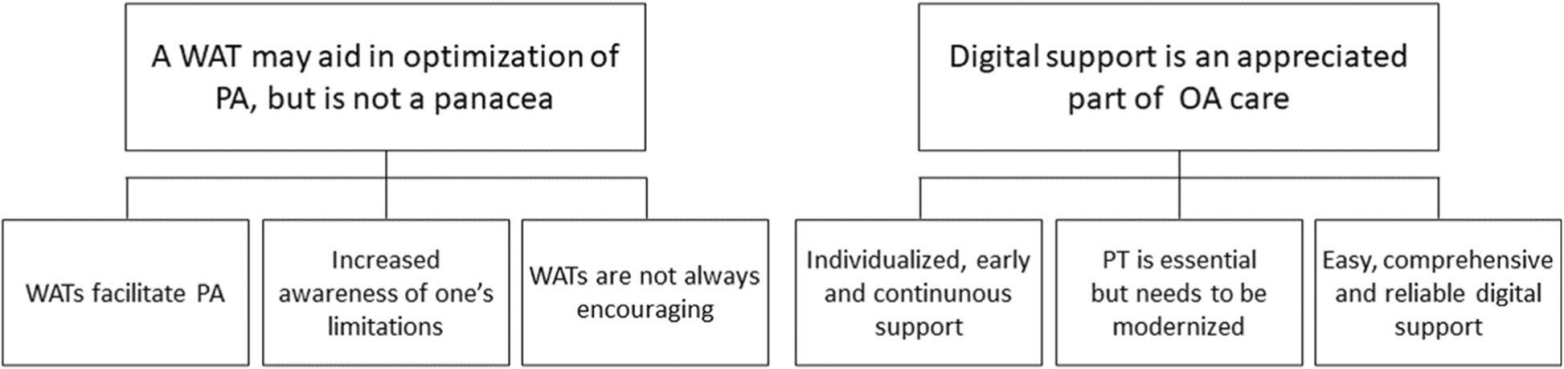
Main categories and sub-categories

Two main categories were identified during the analyses: A *WAT may aid in optimization of PA but is not a panacea* and *Digital support is an appreciated part of OA care.* The main categories and their subcategories are presented in Fig. 1. Representative quotes from all three focus group discussions are attached to each category.

### A WAT may aid in optimization of PA, but is not a panacea

The participants expressed that the WAT in different ways had facilitated PA and increased their awareness of the number of steps that were optimal for handling their OA symptoms. However, using the WAT was not experienced as encouraging for all participants and in some situations, prompts from the app regarding PA were experienced as stressing and discouraging if they were unable to walk.

#### WATs facilitate PA

The WATs facilitated PA in more than one way. Targeting and reaching the daily step goal were experienced as a spur to walk more than usual. The participants described that they would walk around the block or take the dog out for an extra walk in the evening if they saw that they were some steps short of reaching the goal. To set a realistic and achievable step goal and to have a “good enough is perfect” approach when it came to doing PA were seen as important.

*“...it will be easy to push or trigger yourself to go those steps extra if you are at 6,500, it is easy to motivate and take another walk to reach the goal.”*

*Quote from discussion 1*

*“I’m amazed at how controlled I am by it, 7,000 steps, it was like, that's what I walked every day. And now that I don’t have this [the WAT] anymore, I don’t think I take that many steps anymore. I'm really affected by it.”*

*Quote from discussion 2*

The different feedback from the Fitbit app (prompts, reminders, and rewards) were also experienced as an incentive to do more PA, especially walking. They could receive prompts about reaching the step goal but also reminders to move if they had been sedentary for some time.

“It’s positive that it beeps when you haven’t walked 250 steps in an hour. When it “beeps” you get to move and take a turn in the corridors at work…”

*Quote from discussion 1*

#### Increased awareness of one’s limitations

One aspect that surfaced during the discussions was that the WATs not only facilitated PA but also made the participants aware of their PA level and their limitations in engaging in PA, especially walking longer distances. They used information about the number of steps taken and related it with their pain, and other health-related issues. In that way, they became more aware of the number of steps that were optimal specifically for themselves. When they stayed within their optimal number of steps, they experienced less pain flares and less pain-related disruptions of their regular exercise. However, sometimes, the reason for a pain flare was unknown. Some participants could not identify any pattern at all regarding PA and pain.

*“- And that you learn the relationship with how you feel. - Yes, exactly. - The leg or the knee or the hip or whatever it is... That you learn how many steps I must walk so that it does not hurt.”*

*Quote from discussion 1*

*“We had it for so long that I felt that at 8,000 [steps] it started to get too tough afterwards, so I tried to stick to it, and I thought it worked well. Then there was never any [pain]... So previously I activated myself a lot and then nothing... It became a much better rhythm.”*

*Quote from discussion 3*

To be able to show others how many steps they had walked during the day was also seen as valuable. It could be used as a sort of evidence and legitimate their need for rest whether it is after a day at work or after an entire day of sightseeing while on vacation.

*“I could see [in the app] that I should probably quit now. Plus, you can say it to others: I have taken the steps that I can manage, and I can’t tag along any longer.”*

*Quote from discussion 2*

#### WATs are not always encouraging

Both limitations and disadvantages of using a WAT were highlighted during the discussions. Some speculated that the facilitating effect of the WAT depended on the interest of the user. A WAT and the information/feedback serves no purpose if the user is not encouraged by it. Another factor that could limit the facilitating effect was if the user was hindered in walking because of OA pain or functional limitations. Also, those already being highly physically active expressed limited effects of the WAT since there was limited room for increasing their PA.

*“It is not a purpose in itself to have a digital app, you must be spurred by it as well. So just putting on a Fitbit does not help if you are not interested.”*

*Quote from discussion 1*

“So, unfortunately, it had no effect because I [my hip] is so terribly bad and consequently, I could not walk as much as I would like.”

*Quote from discussion 2*

Concerns and experiences of anxiety and stress related to WAT-use were expressed in the discussions. These feelings were experienced when they failed to reach their step goal, when they received prompts from the WAT to move but was not able to walk due to driving a car, attending a meeting etc. To push oneself too hard and never feel content with the amount of PA was also highlighted as a disadvantage of WAT-use.

“You get disquiet if you do not reach 7,000 steps… I think it happened to me one day and that was very tough…”

*Quote from discussion 1*

*“You can go too far with this, as you said, you push yourself and then you have to do a little more and then you have to do a little more and you will never be satisfied.”*

*Quote from discussion 1*

### Digital support is an appreciated part of OA care

Digital support in OA care was, in general, discussed in positive terms but a combination of traditional face-to-face OA care and digital support was perceived as the best solution. Perceptions on OA care, functionality of digital support and the PTs’ role were also highlighted.

#### Individualized, early, and continuous support

It was considered important that the advice and exercise delivered in OA care were individualized and that the health care personnel or personal trainer identified what would motivate individuals to engage in PA. They also felt that the traditional care failed to recognize that also younger, working individuals are affected by OA. The SOASPs are often held during working hours and some participants had experienced that the attendees of OA were mostly older individuals.

“I feel that this… I participated in the SOASP… that it was me and then it was 90-year-olds.”

*Quote from discussion 3*

*“It [SOASP] should be sort of more separated in the age groups maybe because I have no one... but it felt like they were not in the same stage as I was. I would probably like to have that.”*

*Quote from discussion 3*

The timing of OA care was also discussed. They would like to have received information and treatment at an earlier stage of the disease. Self-monitoring certain aspects of one’s own health and detecting any changes was seen as a way to encourage seeking care at an early stage. More frequent visits to health care personnel in the early stage of the disease was also mentioned to support and consolidate behavior change or learn suggested exercises. The need for continuous support from health care was also stressed among the participants. A suggestion emerged of an OA-PT that would see them regularly for check-ups. The suggestion was based on their experiences of individuals with diabetes that see a nurse specialized in diabetes for check-ups once yearly.

*“Early, yes. So that you can come to this realization about losing weight and that you need to train certain things or so. Otherwise, it’s just; ‘Well, now I have a little worse mobility, now I lean forward…’ ”*

*Quote from discussion 3*

*“.. you have a mentor or a physiotherapist that you meet every three months or when necessary to update your exercises, steps, and the Fitbit. Help with that…Find the level, get that support. – Kind of like a diabetes nurse.*

*- Yes, it might be like that.—OA physiotherapist.”*

*Quote from discussion 2*

#### PT is essential but needs to be modernized

PT has a key role in OA care, both traditional and digital care. Some of the participants had experiences of a digital platform for OA care. They appreciated that they had a personal and continuous connection with a PT in the digital platform that could individualize their exercises and offer guidance and support. One functionality that they lacked in the digital platform was the possibility to receive feedback regarding how they performed their exercises. They received the exercises on video but could not film themselves and show the PT.

*“That's what I miss about Joint Academy* (digital platform). *I have never shown how I do my exercises. So theoretically, I can do them completely wrong.”*

*Quote from discussion 1*

The participants talked about sharing their WAT activity information with a PT. A positive aspect was that the PT would gain more information regarding their health and would therefore be able to guide them better regarding PA, exercises etc. Knowing that there was a recipient to their activity data was also seen as a motivating factor. To trust the PT that they shared their activity information with was important.

*“Someone could help me check what it is that makes me feel so bad today, if it’s because I did too much or I did too little or what could be the cause... Then I was grateful because I can’t find a pattern myself and don’t really know...”*

*Quote from discussion 2*

“It can be a good discussion basis for the follow-up visit: “You have walked far too much” or “you have not moved enough.””

*Quote from discussion 3*

PT treatment was discussed in the three sessions and particularly home exercises with stick figure drawings on paper. The participants did not appreciate that they received stick figures drawn by their PT. To instead be provided with instructional videos of the exercises was seen as a superior alternative compared to exercises illustrated with stick figures.

“The stick figures should have been a video instead.—Yes, an instructional film.”

*Quote from discussion 1*

#### Digital support should be easy, comprehensive, and reliable

High availability, more frequent feedback, and initial help with setting up the app or WAT were mentioned when digital support was discussed. There were diverging opinions about apps and WATs in general. Where some participants expressed a great interest in them and had many apps in their smartphone, others said that they had no general interest in apps or WATS and that they wanted a simple support that worked as intended.

“I think it’s a problem, that you can’t get in… That I can’t make it work. I feel it’s like a sort of handicap. But once it works, it's amazing.”

*Quote from discussion 2*

*“… someone must probably instruct me what to do and how to set it up because as I said, I’m not interested in sitting and looking among the apps and what features they have and so on...”*

*Quote from discussion 3*

Desired features of digital support were brought up in the discussions. They appreciated step counting, feedback, and reminders to move that existed in the Fitbit. Other desired features of an optimal digital support were information, automatic registration of PA, to receive new exercises (on video) automatically, reminders to do the exercises and to be able to check it off from a list when you have finished an exercise. A more comprehensive digital support was also discussed with additional features supporting weight loss (logging food and counting calories).

“I would like to have an increased support so that you get the whole concept of diet and other things as well, it would have been great, I think.”

*Quote from discussion 3*

Experiences related to the reliability of the measurements and the data security of the WAT also surfaced during the discussions. A fear that unauthorized individuals or organizations would get access to the users WAT-data was also mentioned as a disadvantage, but they didn’t feel it was a major issue for them. Also experiences about the accuracy of the WAT were discussed. The WAT did not measure all PA which was seen as a limitation. Participants had also experienced that the WAT sometimes measured incorrectly, registering other activities as steps, or not registering other activities at all.

*“It was very often that I looked at [the WAT] and... Oh, ok, so now I have cycled for twenty minutes at a high pace, and I received no credit for it. It’s annoying.”*

*Quote from discussion 1*

## Discussion

This focus group study reports the experiences and perceptions of WAT-use and digital support in working individuals with hip and knee OA. Experiences of the WAT as a tool to facilitate and optimize PA emerged in the discussions but also diverging experiences and perceptions were described; WATs could be discouraging for some individuals and in certain situations. Digital support was perceived as a valuable part of OA care and the participants perceived that it should be individualized, easy, continuous, and reliable. The categories can also be linked to behavior change techniques such as self*-monitoring of behavior, social support, problem solving* and *goal setting* [17]*.*

Although WAT-use in interventions to promote PA is a relatively new phenomenon, there has been a rapid increase in its popularity and use in research during the last decade [21]. Several meta-analyses have reported that WAT-use seems to increase PA in different populations [55]. The experiences of the WAT as a tool to facilitate PA is also reported in a US study describing and comparing current and former WAT-users where a majority (both current and former users) answered that the device influenced increased PA [56]. Correspondingly, a qualitative study reported that patients with OA or inflammatory arthritis described that the WAT reinforced their motivation and helped them to reach their activity goal [36]. The importance of having a step goal to strive for is also shown in other qualitative studies reporting experiences from individuals with OA, arthritis, and type 2 diabetes [36, 42, 57]. The participants in this study also experienced that the WAT made them aware of how many steps per day that was optimal for them to avoid worsening of pain. This experience that both too *little* and too *much* PA might be suboptimal in OA has been described as a U-shaped relationship [58, 59]. WAT-use may aid the individual in finding the PA dosage that works best for them. In line with this, clinicians in the study by Leese et al. [36] expressed that the WAT could work as a “teaching tool” to help patients with OA and arthritis see the connection between the level of PA and the perceived pain.

Negative opinions and limitations of WAT-use were also highlighted in the discussions. They perceived that WATs would be more encouraging if the user had at least some interest in technology. This is in line with the results from a US study describing and comparing current and former WAT-users [56]. That study reported that the top three reasons for WAT-use (current and former users) were ‘an interest in the technology’, ‘to monitor health variables’ and ‘aid to lose weight’. Even the interested and positive WAT-users in this study expressed that there were situations in which the WAT gave rise to feeling more discouraged or irritated than encouraged. They could feel discouraged when they were in so much pain that they could not walk enough to reach their step goal. These feelings of discouragement when using a WAT are reported also in previous research [36, 60]. In the study by Leese et al. [36], both patients and rehabilitation professionals expressed that the WAT-user might feel discouraged and uninspired by the activity information from the WAT if they could not reach their goal due to a fluctuating ability to walk or a constant deterioration. This could possibly be avoided if individual and realistic goals are set together with a rehabilitation professional instead of only using the default goals of the WAT-app [36, 61].

The other main category in this study entailed participants’ experiences and perceptions of digital support in OA care. In general, the participants talked about digital support in positive terms. Having digital support was seen as accessible and could help them to easily gain more knowledge regarding their disorder and their health. These results are also reported in previous research where patients with OA described that having more information of their disorder and health would empower them to manage their symptoms better [38]. The participants in that study also expressed that if they could share data from their WAT with a health care professional, their information would be more objective and accurate. The health care professional would then have more knowledge and be able to make more informed and individually targeted recommendations. To share activity information with others might increase the adherence to WAT-use [62].

In a previous study exploring individuals’ (with OA) perspectives on mHealth, participants expressed that they would appreciate a simple data input, personalized settings, and individual goals in a mHealth app [41]. Other wanted features that were brought up during the discussions in our study were that the OA care and digital support should be early and continuous. An early and continuous care in OA could be important as a preventive measure to reduce the risk of avoidance of activities [63].

The importance of PTs in traditional and digital OA care was also discussed. Some of the participants had used a digital platform for OA care and said that they appreciated having contact with a PT through the platform and to receive individualized exercises with video instructions. In a previous study on OA patients’ experiences of an exercise app, the participants said that they needed input from a professional that could see if they were doing their exercises correctly [64]. This was echoed in this study where participants expressed that the optimal OA care would be a hybrid between digital and traditional OA care with physical meetings with their PT. In the study by Danbjörg et al. [64], a combination between digital support and physical meetings was also preferred. When discussing the importance of PT in this study, stick figures illustrating exercises on paper generated lively discussions among the participants. They found the stick figures difficult to interpret and would have preferred to receive the exercises on video instead.

Wanted features of digital support have been reported in previous qualitative studies and were also discussed in this study. Simplicity and comprehensiveness were highly valued in an eHealth intervention [65] while easy, comprehensive, and including several functions such as information about OA and exercises, automatic registration of activity and the ability to log food were wanted features in this study. It was also seen as essential that the digital support worked as intended and was reliable. Previous research reported that users lost interest if the app or other digital support did not function as intended [60].

### Clinical implications

The general results in this study are in line with the results of previous research exploring the experience and perceptions of mHealth and activity monitoring among individuals with hip and knee OA and other musculoskeletal disorders [65]. This strengthens our beliefs that the results from this study can be applied to similar populations. WATs can facilitate PA in different populations but may also be used to guide individuals with OA to find the specific dose of PA that is optimal for them. Pain is often a limiting factor and important to take into consideration when setting a PA goal. The implications of finding the optimal dose of PA are however limited by the WAT used in this study that mainly was used for counting steps. There may be situations where perhaps only bicycling is suitable. Where applicable, a treating PT or other health professional may also receive relevant activity information from the WAT. However, to our knowledge, patients cannot digitally share activity data with a PT in primary health care in Sweden at present. Future health care systems could be constructed to allow for activity- and other health data to be shared to aid the clinician in their recommendations. WATs in general may perhaps facilitate PA particularly for individuals that are physically active already and have an interest in digital support, but some factors which emerged in this study might enhance the possibility to encourage even those that are not as interested.

Within the scope of this study was also the participants’ perceptions of mHealth and digital support in OA care. Digital support was seen as useful and accessible, especially as a complement or part of the traditional OA care with physical visits. Digital health care could probably be used by traditional health care to a larger extent.

In the section below, we present the key clinical implications and suggestions from the results in this study. Some of the implications are somewhat outside the scope of this study but are included since they emerged during the discussions and were seen as relevant.

- When initiating WAT-use, technical “hands-on” support with settings and goals might be needed. Achievable and individualized step- or activity goals are essential.
- Sharing the activity data with a PT or others may facilitate PA and adherence to WAT-use.
- The participants expressed that core treatment in OA should be delivered at an early stage of the disorder.
- The SOASP may need adjustment to suit younger and working individuals.
- Since OA is a chronic disease, OA care should be continuous. The care could be mainly digital but with visits at regular intervals, for example, annually.

### Strengths and limitations

Measures to achieve trustworthiness as suggested by Graneheim and Lundman [66] have been considered throughout this study. A questioning route was used in all three focus group discussions and no alterations were made to this. It was also the same moderator, place, time of day and the discussions took place within a period of a few weeks. An experienced assistant moderator participated in the discussions. Having these contextual factors consistent for all discussions increased the *dependability* of the results. *Credibility* has been strengthened by choosing the most suitable meaning units and presenting the analysis process thoroughly for transparency. Also, quotes from the participants were chosen to represent the content of the discussions. A continuous dialogue between EÖ and KS were held throughout the analysis process to make sure that all data was included in the results. Agreement was continuously sought between the two researchers in the analysis process. After each focus group discussion, the assistant summed up the discussions and offered the participants the possibility to comment*.* We believe that the results in this study could be transferred to a similar population among individuals with hip and knee OA in working age who are probably somewhat interested in mHealth and digital support. Even though many of the study participants were moderate to highly physically active, also participants having low PA levels are represented in this study. Participant characteristics were presented to increase the opportunity for comparison with other study populations.

This study also has limitations. The moderator and first author (EÖ) had met with all participants at least once. The number of meetings and the reason for the meeting(s) differed for each participant, (handing out the Fitbit and lecturing the SOASP). This previous contact might have had an inhibitory effect on the participants’ willingness to talk freely during the discussions. However, since the questions in the discussions were not directly related to their contact with E.Ö, we believe that the participants felt that they could speak freely. The participants in this study are probably not representative of the general population with hip and knee OA, which may have affected the transferability to the general OA-population. Based on data previously collected in the C-RCT, about 40% of the participants in the C-RCT already used a WAT when they registered for the study. This could indicate an interest in WATs and mHealth and might have introduced a selection bias.

Most of the participants were women (72%) which could have had an impact on the results. Previous studies have shown that WAT-use is more common in women [67] and that women have higher adherence to WAT-use in a PA intervention than men [68]. Hence, the participants in our study were perhaps more positive to WAT-use than a sample with an equal sex distribution would have been. Our sample are in other aspects probably similar to individuals participating in SOASPs in Sweden where a majority is women and have OA in the knee.

In this study, 18 individuals agreed to participate which resulted in the three focus groups. Additional participants and a fourth focus group could possibly have provided additional information but given the consistency of the experiences and perceptions in the three discussions, we do not believe that a fourth focus group would have induced any major changes in the results.

## Conclusion

This study provides information on how individuals with hip and knee OA experience and perceive PA monitoring and digital support in OA care. Using WATs may aid in facilitating PA for some individuals but not all. WATs could also help individuals with OA to relate their steps taken or PA conducted to their perceived pain or other health outcomes. This may help them (and their PT) to optimize the PA level. Digital support was seen as an appreciated part of OA care but preferably, it should be a hybrid solution between traditional OA care and digital OA care. Health care should offer solutions for a hybrid health care that is individualized, comprehensive, easy, reliable, and continuous.

## Supplementary Information

**Additional file 1.** Questioning route.

**Additional file 2.** COREQ.

## Data Availability

The data in this study is based on transcribed discussions with the participants and can therefore not be entirely anonymized despite that the names have been removed. Hence, there is a risk that the participants could be recognized. However, reasonable requests to access the data should be made to the corresponding author.

## Abbreviations

OA: Osteoarthritis
PA: Physical activity
WHO: World Health Organization
mHealth: Mobile Health
WAT: Wearable activity tracker
App: Application
COREQ: Consolidated criteria for reporting qualitative research
C-RCT: Cluster-randomized controlled trial
SOASP: Supported osteoarthritis self-management program
EÖ: Elin Östlind
KS: Kjerstin Stigmar
EEH: Eva Ekvall Hansson
PT: Physiotherapist; physiotherapy
SD: Standard Deviation
IPAQ-SF: International physical activity questionnaire- Short form
