# Supplementary material for: Experiences of activity monitoring and perceptions of digital support among working individuals with hip and knee osteoarthritis – a focus group study
Source: BMC Public Health. 2022 Aug 30;22:1641. doi: 10.1186/s12889-022-14065-0 (PMC9426251; doi:10.1186/s12889-022-14065-0)
Supplement: Supplementary file 1 — Additional file 1. Questioning route. [file 12889_2022_14065_MOESM1_ESM.docx]

# Focus group discussion – question route

## Introduction

The moderator introduces herself and the assistant, introduces the topic, briefly explains that we want to take part of the participants' experiences of monitoring their activity with a Fitbit and their general perception of digital support for osteoarthritis. All participants are asked to let the information that emerges from this discussion stay in the group so that everyone feels safe talking freely. If someone should feel worse after the discussion, that person is recommended to begin with contacting their Health Care Center. There are no right or wrong answers, we would like to take part of everyone's experience and perception on this topic. We use tape recorders to record what is said during the discussion, so try to speak one at a time. Please speak firstly to each other instead of to the moderator. Feel free to talk to each other in the first place and not to the moderator.

Opening question

One participant at a time may introduce themselves and answer (briefly) to the question:

- Have you used any health or exercise apps, and if so, which ones?

Introductory questions

- What are your general thoughts on the concept “digital support” in relation to osteoarthritis?
- What are your experiences of the intervention that you have taken part in, i.e., to monitor (follow) your activity with a Fitbit? Pros/cons?

Key questions

- In what way (if any) would you say that the Fitbit has affected your physical activity? How/why?
- What kind of (digital) support would you like to have in dealing with osteoarthritis in everyday life?
- What are your feelings regarding digital healthcare or self-care, replacing some of traditional healthcare? Pros/cons?
- How would you feel about sharing your activity information with, for example, your physiotherapist?
- What would you like the osteoarthritis treatment of the future to look like?
- A digital support for osteoarthritis - is it something that you would consider paying for?

Ending questions

(The assistant summarizes the questions and what topics have come up during discussion in general).

- Is there something you would like to add?

Debriefing afterwards

Discussion between moderator and assistant (being recorded). First impressions of the discussion, main issues and overall impressions. Thoughts for the next discussion?
